# TTBK2: A Tau Protein Kinase beyond Tau Phosphorylation

**DOI:** 10.1155/2015/575170

**Published:** 2015-04-09

**Authors:** Jung-Chi Liao, T. Tony Yang, Rueyhung Roc Weng, Ching-Te Kuo, Chih-Wei Chang

**Affiliations:** Institute of Atomic and Molecular Sciences, Academia Sinica, No. 1, Roosevelt Road, Section 4, Taipei 10617, Taiwan

## Abstract

Tau tubulin kinase 2 (TTBK2) is a kinase known to phosphorylate tau and tubulin. It has recently drawn much attention due to its involvement in multiple important cellular processes. Here, we review the current understanding of TTBK2, including its sequence, structure, binding sites, phosphorylation substrates, and cellular processes involved. TTBK2 possesses a casein kinase 1 (CK1) kinase domain followed by a ~900 amino acid segment, potentially responsible for its localization and substrate recruitment. It is known to bind to CEP164, a centriolar protein, and EB1, a microtubule plus-end tracking protein. In addition to autophosphorylation, known phosphorylation substrates of TTBK2 include tau, tubulin, CEP164, CEP97, and TDP-43, a neurodegeneration-associated protein. Mutations of TTBK2 are associated with spinocerebellar ataxia type 11. In addition, TTBK2 is essential for regulating the growth of axonemal microtubules in ciliogenesis. It also plays roles in resistance of cancer target therapies and in regulating glucose and GABA transport. Reported sites of TTBK2 localization include the centriole/basal body, the midbody, and possibly the mitotic spindles. Together, TTBK2 is a multifunctional kinase involved in important cellular processes and demands augmented efforts in investigating its functions.

## 1. Introduction

Tau is a microtubule-associated protein (MAP) responsible for stabilizing microtubules [1, 2]. It is abundantly expressed in neurons and plays an important role in neuronal cytoskeleton stabilization [3–6]. The structure of tau can be divided into four segments: the N-terminal region, the proline-rich domain, the three- or four-repeat microtubule binding domain (MBD), and the C-terminal region [7–9]. There are more than 85 phosphorylation sites on tau distributed along the four segments [10, 11]. Each of the repeat regions contains a conserved motif KXGS, where the serine residue can be phosphorylated, destabilizing the microtubules in neurons [2, 12, 13]. Phosphorylation of sites in other regions of tau has different levels of impact on microtubule stability [10, 14].

Specifically, hyperphosphorylation of tau is a signature of Alzheimer's disease (AD) [6, 15, 16]. Among the known phosphorylation sites, Y18, S68, T69, T71, S113, T123, T153, T175, T184, S185, S191, and Y197 in the N-terminal region, S208, S210, S214, S237, and S238 in the proline-rich domain, S258, S262, S289, and S356 in the microtubule binding domain as well as Y394, T403, S409, S422, T427, S433, and S435 in the C-terminal region (numbered in isoform 2 of tau, 441 amino acids) are phosphorylated in AD brains but not in normal brains [10, 17]. Hyperphosphorylation of tau results in abnormal aggregation of tau and reduces its affinity to microtubules, destructing tau-associated cellular activities such as axonal growth, vesicle transport, and signal propagation mediated by microtubules [18, 19]. These effects may potentially be implicated in symptoms of AD.

More than 20 kinases can phosphorylate tau, where most of these kinases are involved in phosphorylating AD sites of tau [17, 20]. Tau protein kinases have been grouped into three categories: (1) proline-directed protein kinases, including GSK3, CDK5, MAPK; (2) tyrosine protein kinases, including Src family kinase and c-Abl; and (3) other protein kinases, including TTBK1/2, CK1/2, DYRK1A/2, MARK, PKA, PKB, PKC, and PKN [17]. Each kinase has specific phosphorylation sites and is involved in neurodegeneration associated with AD.

TTBK1 and TTBK2 contain a highly homologous kinase domain, with 88% identity and 96% similarity (residues 35 to 294 in TTBK1 and residues 21 to 280 in TTBK2) [21, 22]. The majority of the sequences outside of this domain are different between these two kinases, with the exception of segments of 1053 to 1117 in TTBK1 and 942 to 1006 in TTBK2. TTBK1 is highly expressed in cortical neurons while TTBK2 is highly expressed in the cerebellum Purkinje cells, the granular cell layer, the hippocampus, the midbrain, and the substantia nigra [22]. TTBK1/2 can phosphorylate tau at 10 different sites, all of them associated with AD [23, 24]. Both TTBK1 and TTBK2 belong to the casein kinase 1 (CK1) superfamily [21–23].

TTBK1 is upregulated in AD brains and it phosphorylates tau at the sites found in paired helical filaments [15, 23]. It is responsible for neurofibrillary pretangle formation due to tau phosphorylation at Ser422 in neurons [25–27] and subsequent tau aggregation. TTBK1 has also been found to be associated with late onset AD [28]. Notably, the crystal structure of the TTBK1 kinase domain has previously been determined [29, 30].

**Figure 1:**
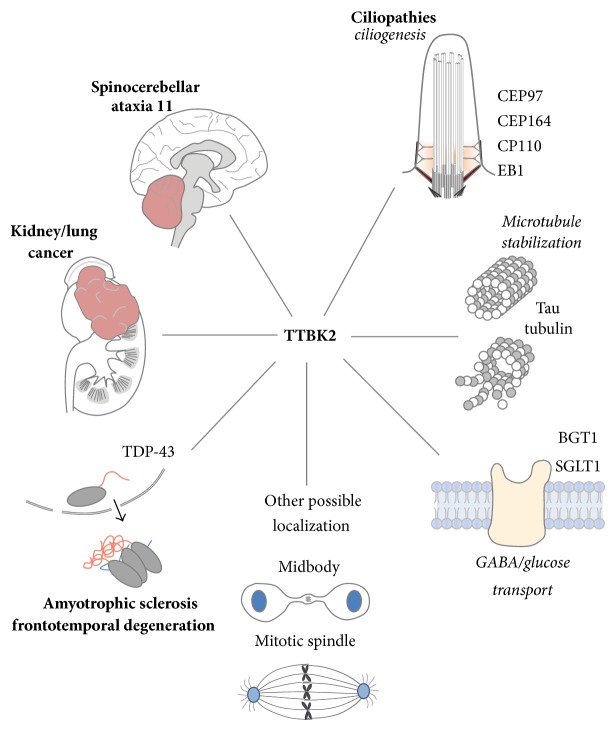
Known cellular processes and diseases associated with TTBK2. TTBK2 is involved in tau/tubulin phosphorylation, ciliogenesis, SCA11, cancer progression, transporter stimulation, and TDP-43 accumulation, among other processes. Diseases are shown in bold fonts and cellular processes are shown in light face italic fonts.

TTBK2 is another serine/threonine protein kinase of the CK1 superfamily that is able to phosphorylate both tau and tubulin [31–33]. Two specific phosphorylation sites on tau have been identified, that is, S208 and S210 (numbered in isoform 2), both associated with AD [10, 32]. It has also been found that the preferred substrate of TTBK2 possesses a phosphotyrosine at the +2 site [34], although neither S208 nor S210 of tau has a tyrosine at the +2 position. TTBK2 is expressed in multiple types of tissues, including the liver, kidney, heart, pancreas, skeletal muscle, and in particular the brain, where it is largely found in the cerebellum [22, 31, 32]. Although TTBK2 can phosphorylate two AD-associated sites of tau, the major phenotype of mutant TTBK2 is not associated with AD but with autosomal dominant spinocerebellar ataxia type 11 (SCA11), a type of serious neurodegeneration [22]. The disease mechanism linking TTBK2 mutation and SCA11 remains unclear. In addition, TTBK2 was found to be an essential kinase for ciliogenesis initiation [35]. TTBK2 has also been implicated in cancer progression [36, 37], transporter stimulation [38, 39], and TDP-43 accumulation [40], among other processes. Known cellular processes and diseases associated with TTBK2 are summarized in Figure 1. Therefore, the role of TTBK2 does not appear to be restricted to regulation of microtubule stability in neurons. The underlying mechanisms of these involved diseases, including SCA11 and cancer, as well as cellular processes including ciliogenesis, remain largely elusive. Here, we review TTBK2's structure and its involvement in spinocerebellar ataxia, ciliogenesis, cancer, and other cellular activities.

## 2. The Structure of TTBK2

**Figure 2:**
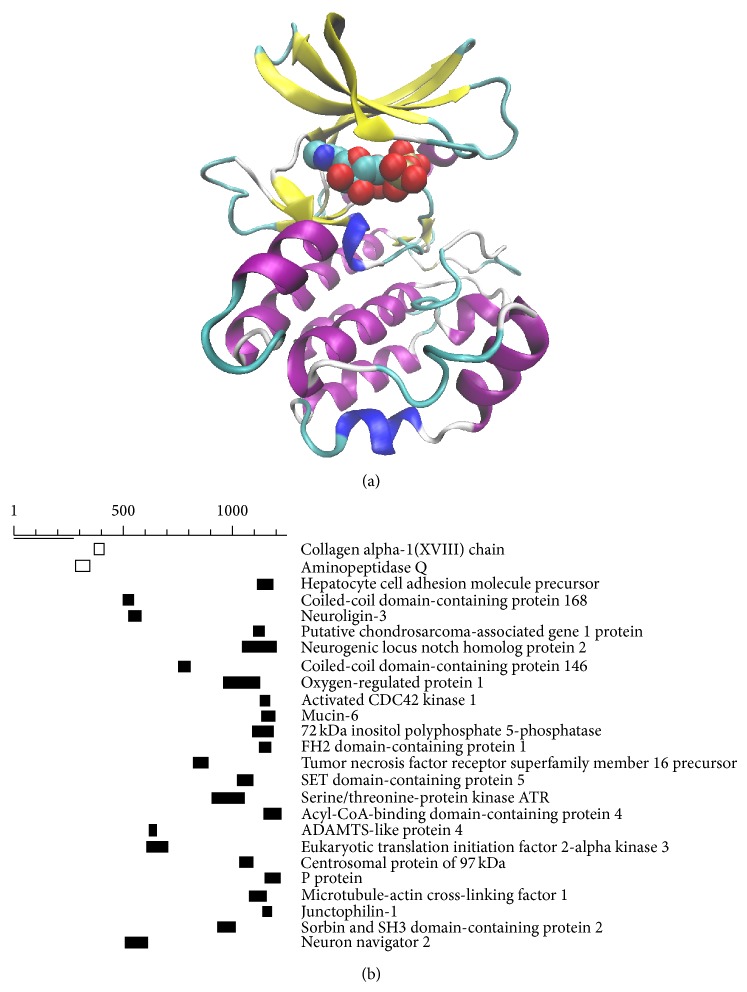
Structure and sequence analysis of TTBK2. (a) A model structure of the kinase domain of TTBK2 with a bound ATP using the crystal structure of TTBK1 as a template. (b) Top-hit homologous sequences of the noncatalytic domain of TTBK2 identified by protein BLAST. Two separate BLAST searches were performed against two segments: residues 281 to 448 covering the region remained in SCA11 patients (white bars) and residues 489 to 1244 covering the rest of the noncatalytic domain (black bars).

The human TTBK2 protein is composed of 1244 amino acids where residues 21 to 280 form its kinase domain. A crystal of the N-terminus of human TTBK2 has been obtained, although the coordinates were not reported [41]. Using the structure of TTBK1 complexed with ATP (PDB: 4BTJ) as a template [29], we created a model structure of TTBK2's kinase domain (unpublished) using SWISS-MODEL [42, 43], as shown in Figure 2(a) (GMQE: 0.96, GMEAN4: −0.19). The structure is highly similar to the one for TTBK1. TTBK1 and TTBK2 possess a P-P-E motif in the region VIII of the kinase domain, different from that of other kinases [34, 44].

The C-terminal beyond the kinase domain of TTBK2 is largely unexplored in terms of its structure and function, except for the presence of a short SxIP motif previously reported [45]. This region of ~960 residues beyond the kinase domain should be responsible for kinase localization and substrate recruitment. Phylogenetic profiling and protein BLAST search using the full protein length show that not only the kinase domain but also the noncatalytic domain are highly conserved among species (data not shown). We performed a BLAST search of the noncatalytic domain, splitting it into two segments: residues 281 to 448 covering the region remained in the gene of SCA11 patients and residues 489 to 1244, against the human RefSeq database. The BLAST results after removing redundant hits (isoforms of the same protein) are shown in Figure 2(b) and Table 1 (unpublished). Because the sequence identity is relatively low (~30%), it is likely that some of these BLAST results are false positive. Nonetheless, as finding remote homologs of “orphan” proteins in other studies [46], these results suggest testable hypotheses for potential binding or functional sites of TTBK2. For the segment of residues 281–448, we found two potential homologs: collagen alpha-1(XVIII) chain and aminopeptidase Q. For the segment of residues 449–1244, several hits were found after relaxing the statistical significance threshold (increased to 100), especially toward the C-terminus. Among the homologous proteins shown in Figure 2(b) and Table 1, several neuron-associated proteins are present, for example, neuroligin-3 and neurogenic locus notch homolog protein 2. It is known that TTBK2 is highly expressed in neurons, so these regions with homologous sequences to other neuron-associated proteins may provide hints of possible structural elements involved in neuronal localization of TTBK2. CEP97 is known to be a key negative regulator of ciliogenesis in collaboration with CP110 by capping the mother centriole [47], indicating possible links to TTBK2's roles in ciliogenesis initiation (see below).

## 3. Spinocerebellar Ataxia Type 11

SCA is a progressive genetic disease with several types associated with a variety of mutations [48]. SCA11 is one type of SCA caused solely by mutation of TTBK2 [22]. Four family cases from Germany, French, Pakistan, and England have been reported to have SCA11 [22, 49, 50]. All four cases find similar single heterozygous truncated TTBK2 (i.e., TTBK2^truncated/+^) caused by a premature stop codon and termination at residues 448 (frameshift deletions at codons 435), 448 (frameshift deletions at codons 435), 449 (frameshift deletions at codons 428/429), and 450 (one base insertion at codon 444), respectively. As shown above, the serine-rich segment between the kinase domain end and residue 448 contains aminopeptidase Q and collagen alpha-1(XVIII) sequences (Figure 2(b) and Table 1). Patients with SCA11 exhibit symptoms of movement disorder indicative of progressive cerebellar ataxia and abnormal visions, but their life expectancy remains similar to that of the general population [22]. These symptoms are different from those of ciliopathies, suggesting differential roles of TTBK2 involved in cells. The function of the segment homologous to part of aminopeptidase Q and collagen XVIII beyond the kinase domain remains to be explored. For mouse, TTBK2^fmly1/fmly1^ mice with homozygous mutants of truncated TTBK2 at residue 450 were embryonic lethal at embryonic day 10, with indistinct brain subdivisions, distorted caudal bodies, and delayed body and brain development [34]. Heterozygous mutant TTBK2^fmly1/+^ mice were completely normal and exhibited a regular lifespan.

## 4. TTBK2 and Ciliogenesis

The primary cilium mediates multiple signaling activities including sonic hedgehog signaling, noncanonical Wnt signaling, calcium signaling, and PDGF*α* signaling [51–55]. It possesses a patterned distribution of microtubule doublets, mostly in a 9+0 ringed arrangement, extended from the mother centriole-derived basal body, allowing active transport by kinesin and dynein along these microtubule tracks. A mature primary cilium is about 3 to 10 *μ*m in length. The formation and the resorption of these ring-patterned microtubule doublets, or the axoneme, are closely regulated according to the phase of the cell cycle as well as other cellular activities [56–59]. When a cell is in the quiescent state, a primary cilium extends its axoneme originating from the 9 microtubule triplets of its basal body and reaches its equilibrium length. During the ciliogenesis process, tubulin precursors are transported by intraflagellar transport (IFT) proteins and molecular motors and added to the ciliary tip. At this stage, the cilium is potentially capable of serving its sensory and signaling functions. Before cell cycle reentry, the cilium is resorbed to prepare for centriole duplication. In the ciliary resorption process, the axonemal microtubules are destabilized, resulting in disassembly of axonemal subunits and shortening of the cilium [57, 60–62]. The assembly, maintenance, and disassembly of primary cilia require delicate control of axonemal cytoskeleton remodeling.

Mutations of ciliary proteins result in early embryonic lethality or serious diseases collectively called ciliopathies [63–67]. Ciliopathies include Meckel-Gruber syndrome (MKS), Joubert syndrome (JBTS), nephronophthisis (NPHP), and Bardet-Biedl syndrome (BBS) [63, 68–73], characterized by renal cystic dysplasia, retinal degeneration, postaxial polydactyly, cerebellar ataxia, developmental delay, tubular cysts, tubulointerstitial nephropathy, and truncal obesity [68–70, 74, 75]. Genes and clinical manifestations associated with different ciliopathic syndromes may be found in earlier reviews (e.g., see summarized tables in [66]). Most reported cases of ciliopathies possess homozygous mutations or compound heterozygous mutations of ciliary proteins [76]. MKS is perinatal lethal, the most serious among all ciliopathies, while NPHP is relatively less severe. The phenotypes of a single mutation can vary in severity. For example, different mutations in TMEM67 cause different levels of severity, ranging from mildly nonlethal to lethal [77].

**Figure 3:**
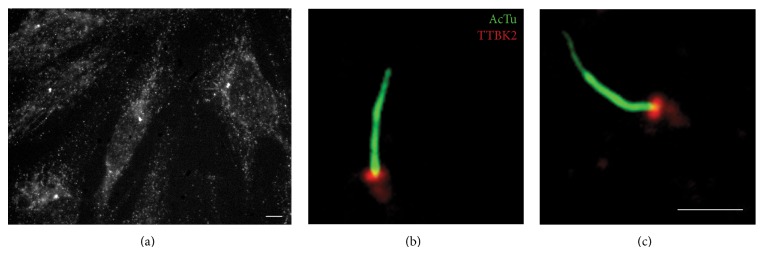
Localization of TTBK2 in cells and close to primary cilia. (a) Signals of TTBK2 could be detected in the cytoplasm, with bright puncta at the base of primary cilia. (b, c) Close to primary cilia, TTBK2 was mostly localized at the distal end of the basal body. Dim signals of TTBK2 could also be seen along the basal body. Scale bars: (a) 5 *μ*m, (b, c) 2 *μ*m.

**Figure 4:**
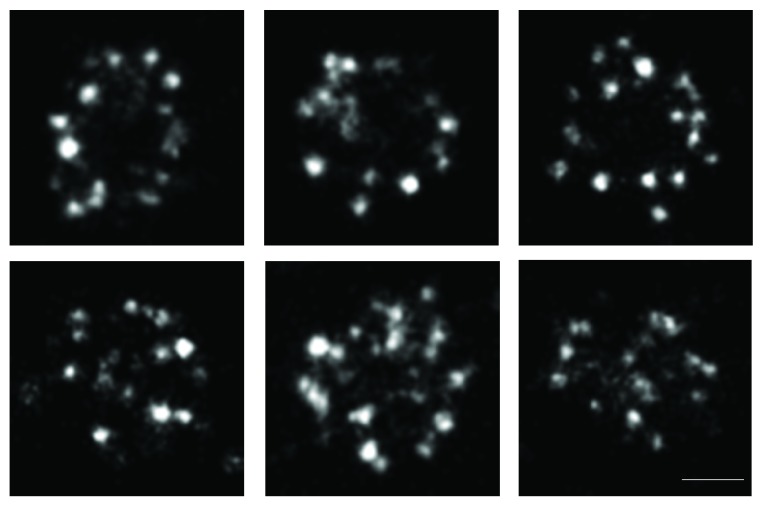
dSTORM superresolution imaging revealing a ring-shaped pattern of TTBK2 presumably at the distal appendages. The annular arrangement has an average diameter of 436 ± 34 nm (*n* = 15), close to the diameter of the tips of distal appendages. Scale bar: 200 nm.

Among many proteins regulating ciliogenesis, TTBK2 has recently been found to play key roles in initiating ciliogenesis [35, 78]. When a cell is in its quiescent state, distal appendages of the mother centriole are docked to a ciliary vesicle. Major components of distal appendages include C2CD3, CEP83, CEP89, SCLT1, FBF1, and CEP164, in a hierarchical order [79, 80]. CEP164 recruits TTBK2, where TTBK2's proline-rich motif serves as the binding site for the WW domain of CEP164, and can then be phosphorylated by TTBK2 [81–83]. The localization of TTBK2 at the base of primary cilia or the distal end of basal bodies can be clearly seen as shown in Figure 3 (unpublished, same as the localization seen by others [35]). A weak localization signal of TTBK2 on the basal body was also observed (Figure 3). TTBK2 mediates the recruitment of IFT complexes and triggers the removal of CP110 [35], which is recruited to the centriole distal end by CEP97 to suppress ciliogenesis by inhibiting microtubule assembly [47]. CEP97 can be recruited to the microtubule plus end by CEP104 [84], a protein possessing the SxIP motif, a motif also found in TTBK2 [45, 81]. Depletion of CP110 and CEP97 results in abnormally long centrioles, implicating these proteins in regulation of microtubule elongation of centrioles [47]. It is found that TTBK2 can phosphorylate CEP97 in vitro [81]. Extending our superresolution studies of primary cilia [85], our unpublished data show that TTBK2 forms an unevenly distributed annular arrangement with a diameter similar to that of the tips of distal appendages as visualized by direct stochastic optical reconstruction microscopy (dSTORM) imaging (Figure 4), where only a small portion of fluorophores are randomly excited and localized to reach ~20 nm resolution. It is thus unclear whether TTBK2 can reach CEP97 in cells at this location and how the subsequent CP110 removal occurs. Our sequence analysis shows a segment of TTBK2 is homologous to CEP97, implying their similarity in a function or a structural motif. For mice with mutant TTBK2 missing part of the kinase domain and all subsequent residues (TTBK2_bby_ truncated at residue 141 within the kinase domain), transition zone proteins such as MKS1 and TMEM67 were found to localize to the ciliary base even though CP110 is present [35], suggesting the TTBK2 pathway may be independent of the biogenesis of the ciliary transition zone. TTBK2 can also bind to EB1 through its SxIP motifs [81], where EB1 is a microtubule plus-end tracking protein required for ciliogenesis, as evidenced by the defect in microtubule minus-end anchoring at the basal body of EB1-depleted cultured cells possessing short cilia [86, 87]. The significance of TTBK2-EB1 interaction during ciliogenesis remains unclear. Thus, TTBK2 has several additional substrates other than tau, although its roles in the biogenesis of cilia are similarly involved in remodeling of the microtubule cytoskeleton.

Although TTBK2 has been implicated in ciliogenesis, the phenotypes caused by single heterozygous mutation of TTBK2 are not exactly characteristic of ciliopathies but instead of SCA11. They are slightly similar to those of mild JBTS, which is usually caused by homozygous mutations, exhibiting ataxia and developmental delay. Homozygous mutations of TTBK2 in mice show serious prenatal lethality with malformation of brain divisions, closer to more severe phenotypes of MKS or JBTS. Thus, ciliary malfunctions may not be enough to explain disease mechanisms behind TTBK2-associated SCA11. It is possible that the major roles of TTBK2 are associated with ciliary functions in neurons, although the effects of its activities on tau phosphorylation and other possible roles in cells may also contribute to phenotypes associated with mutant TTBK2. Mutation of TTBK2 may potentially be more serious than mutations of other proteins involved in ciliopathies because its heterozygous mutation results in SCA at the age as early as of 11 years old [22].

## 5. Roles of TTBK2 in Cancer

TTBK2 has been reported to exhibit differential expression in cancer cells. Upregulated TTBK2 in kidney carcinoma and melanoma cell lines is correlated with resistance of the target therapeutic drug Sunitinib, while knockdown of TTBK2 in these cells increases Sunitinib sensitivity [36]. Reduction of TTBK2 also increases Sunitinib inhibition of cancer cell migration. For lung adenocarcinoma, on the other hand, TTBK2 was expressed relatively more in tissue samples from subjects without recurrence after 5 years than those with recurrence within 3 years [37], suggesting a somewhat different involvement of TTBK2 in lung adenocarcinoma than in kidney carcinoma and melanoma.

## 6. TTBK2 and Transporter Proteins

TTBK2 has also been shown to play roles in regulating activities of membrane transporters, including Na^+^Cl^−^ coupled betaine *γ*-aminobutyric acid (GABA) transporter BGT1 and Na^+^ coupled glucose transporter SGLT1 [38, 39]. Mutation or depletion of TTBK2 reduced BGT1 and SGLT1 stability in the cell membrane and resulted in loss of GABA or glucose transport capacity in* Xenopus* oocytes. One possible explanation of the involvement of TTBK2 in these transporter activities is the significance of primary cilia functions, although other effects due to TTBK2's phosphorylation functions cannot be ruled out.

## 7. Phosphorylation of TDP-43

In addition to tau, tubulin, CEP164, CEP97, and autophosphorylation, TTBK2 has also recently been found to phosphorylate transactive response (TAR) DNA-binding protein 43 [40], or TDP-43, a DNA/RNA binding protein responsible for transcriptional repression, pre-mRNA splicing, and translational regulation [88]. TDP-43 has recently drawn considerable attention because it has been found that hyperphosphorylation-associated aggregation of TDP-43 is a signature of two neurodegenerative diseases: amyotrophic sclerosis (ALS) and frontotemporal lobar degeneration (FTLD-TDP) [89, 90]. Phosphorylation of TDP-43 by both TTBK1 and TTBK2 overexpression resulted in relocalization of TDP-43 from the nucleus to the cytosol [40]. TTBK1/TTBK2 was found to colocalize with phospho-TDP-43 in the frontal cortex of FTLD-TDP patients and in the spinal cord of ALS patients, suggesting that phosphorylation of TDP-43 by both TTBK1 and TTBK2 likely plays a role in progression of these diseases.

## 8. Additional Localization Sites: Midbody, Mitotic Spindle, and Others

A recent study linking ciliopathies to DNA damage response showed that TTBK2 is localized to the midbody between dividing cells [83]. A strong signal of CEP164 was also observed at the midbody, suggesting that CEP164 may again play a role in recruiting TTBK2 to the midbody, although the functions of TTBK2 at the midbody remains elusive. The image also showed the spreading of TTBK2 in the cytoplasm. Another study of the TTBK homolog in* Drosophila*, Asator, showed the localization of the protein to the mitotic spindle and interaction with the spindle matrix protein Megator [91]. The kinase domain of Asator has 78% identity to that of TTBK2, but the segment beyond the kinase domain is composed of sections that are somewhat homologous to TTBK2 and sections considerably different from TTBK2. It is thus possible that Asator recognizes the signature of the phosphorylation sites similar to TTBK2 but has some variations in localization preference. Finally, a SCA11-associated mutation of TTBK2 resulted in localization of the protein to the nucleus [34], an aberrant protein positioning with unknown effects on cells.

## Figures and Tables

**Table 1:** Potential homologs of the TTBK2 noncatalytic region identified by protein BLAST search.

Description	Alignment score	Query cover	*E* value	Identity
Search query: TTBK2 amino acid 281–448				
Collagen alpha-1(XVIII) chain	30.4	29%	2	35%
Aminopeptidase Q	30	41%	2.3	31%
Search query: TTBK2 amino acid 449–1244				
Hepatocyte cell adhesion molecule precursor	33.9	9%	1	33%
Coiled-coil domain-containing protein 168	31.6	6%	7.8	29%
Neuroligin-3	31.6	7%	8.1	33%
Putative chondrosarcoma-associated gene 1 protein	28.9	6%	8.9	39%
Neurogenic locus notch homolog protein 2	31.2	19%	10	23%
Coiled-coil domain-containing protein 146	30.8	7%	11	33%
Oxygen-regulated protein 1	30	20%	25	26%
Activated CDC42 kinase 1	29.6	5%	31	42%
Mucin-6	29.6	8%	31	32%
72 kDa inositol polyphosphate 5-phosphatase	29.3	11%	32	31%
FH2 domain-containing protein 1	29.6	6%	33	38%
Tumor necrosis factor receptor superfamily member 16 precursor	28.9	9%	41	24%
SET domain-containing protein 5	28.9	10%	49	30%
Serine/threonine-protein kinase ATR	28.5	18%	61	23%
Acyl-CoA-binding domain-containing protein 4	28.1	10%	65	29%
ADAMTS-like protein 4	28.5	4%	67	36%
Eukaryotic translation initiation factor 2-alpha kinase 3	28.5	12%	68	28%
Centrosomal protein of 97 kDa	28.5	7%	70	33%
P protein	28.5	9%	72	34%
Microtubule-actin cross-linking factor 1	28.1	10%	80	27%
Junctophilin-1	28.1	5%	81	38%
Sorbin and SH3 domain-containing protein 2	28.1	11%	83	28%
Neuron navigator 2	28.1	13%	96	28%
